# Supplementary material for: piR-hsa-022095 Drives Hypertrophic Scar Formation via KLF11-Dependent Fibroblast Proliferation
Source: Biomedicines. 2025 Dec 2;13(12):2963. doi: 10.3390/biomedicines13122963 (PMC12730285; doi:10.3390/biomedicines13122963)
Supplement: Supplementary file 1 [file biomedicines-13-02963-s001.zip › biomedicines-3909439-supplementary.pdf]

Table S1. List of primers used in qRT-PCR assay and piRNA inhibitors

Genes		Sequence (5'-3')	Accession numbers
KLF11	Forward	TGGAGAGGAAGCGGCATGACA	NM_003597.4
	Reverse	ACGGCAGAGGACTGGAGAACAT	
SMAD7	Forward	ACCTTAGCCGACTCTGCGAACT	NM_005904.5
	Reverse	AGCGGAGGAAGGCACAGCAT	
CMYC	Forward	TCCCTCCACTCGGAAGGAC	NM_002467.6
	Reverse	CTGGTGCATTTTCGGTTGTTG	
β-ACTIN	Forward	CATGTACGTTGCTATCCAGGC	NM_001101.5
	Reverse	CTCCTTAATGTCACGCACGAT	
hsa_piR_022095	Forward	AACGGCTACCGGAGCTGTGT	/
	Reverse	ATCCAGTGCAGGGTCCGAGG	
U6	Forward	CTCGCTTCGGCAGCACA	NR_004394.1
	Reverse	AACGCTTCACGAATTTGCGT	
hsa_piR_021192 inhibitor		ACAAAGGUCUCCUGAAGGACACACCGGUUA	/
hsa_piR_021489 inhibitor		AUUGGCUCCCAGUGUUCUUGACACUUA	/
hsa_piR_022095 inhibitor		GCGUCCACACACCACACAGCUCCGGUA	/
hsa_piR_013486 inhibitor		UUUAUUAGCUAUCAACACUUAGCUUUCCCA	/
NC-inhibitor		CAGUACUUUUGUGUAGUACAA	/
